# “If we survive this, we’ll make it through anything” – Exploration of the COVID-19 pandemic from the perspective of nursing home staff in Hesse, Germany

**DOI:** 10.1186/s12912-025-03916-x

**Published:** 2025-10-21

**Authors:** Loraine Busetto, Anna-Katharina Meurer, Désirée Wyrwich, Katharina Grikscheit, Sandra Ciesek

**Affiliations:** Institute for Medical Virology, Goethe University Frankfurt, Frankfurt University Hospital, Paul-Ehrlich-Str. 40, 60590 Frankfurt am Main, Germany

**Keywords:** COVID-19 pandemic, Nursing homes, Nursing care, Qualitative research

## Abstract

**Background:**

As the COVID-19 pandemic unfolded, nursing homes became one of its most salient settings given rapid and deadly outbreaks. This study aimed to explore the perception of the pandemic after its conclusion and its implications for the future from the perspective of nursing home (NH) staff.

**Methods:**

We used a purposive sampling strategy to conduct semi-structured interviews with 41 members of staff with different professional backgrounds and at different hierarchical levels at three nursing homes in Hesse, Germany. Interviews were analysed using reflexive thematic analysis by Braun and Clarke.

**Results:**

Four main themes were developed: “It’s us against the world” refers to the strong sense of team spirit, mutual understanding and working towards a common purpose. This was often contrasted against the outside world of those who had not lived through the same experience. “Was it the physical or psychological burden? – Both” describes the mutually amplifying co-incidence of a high psychological burden with an exceptionally high workload in combination with critical staff shortages. “Death and dying – but not in these quantities, not in these circumstances” summarises the staff experience of death and dying, both in quantitative terms as well as qualitative terms, e.g. residents dying without their relatives, and staff not being able to support the dying process or mourn the dead. “From absolutely unprecedented to practiced routines” entails the development from a situation in which no one knew what to do, to a situation of experience and expertise. This was often linked to calls for “outsiders” to listen and learn from NHs.

**Conclusions:**

Our findings describe the subjective experience of NH staff working through the pandemic, shedding light on aspects of teamwork, workload and wellbeing, resident deaths and preparedness for future challenges. We would recommend for future research to focus on mixed methods approaches to investigate to what extent in-depth qualitative insights apply in larger samples. As practice implications, it seems relevant to establish realistic pipelines from practice experts to policy makers in inter-pandemic times, and to work towards improving general conditions in NHs as these will form the foundation for the quality of the pandemic response.

**Study registration:**

German Clinical Trial Register, DRKS00030812, registration date 30.12.2022.

**Clinical trial number:**

Not applicable.

## Introduction

The COVID-19 pandemic brought about significant and far-reaching changes in all areas of society including health care provision. Through 2025, approximately, 777 million COVID-19 cases and 7 million COVID-19 deaths worldwide were reported to the World Health Organisation [1]. In Germany, the cases built to approximately 38 million and around 175,000 deaths [1]. As the pandemic unfolded, nursing homes (NHs) became a highly salient setting given their high likelihood of rapid and deadly outbreaks, due to conducive factors such as shared living spaces and a vulnerable patient population with advanced age and pre-existing illnesses [2, 3]. Additionally, workplace rights and protections for staff had to be taken into consideration. Within the context of the pandemic, NH front line workers were faced with increased risks of infection, morbidity and mortality [4, 5].

Given the importance of the NH setting, various studies have explored the experiences of NH residents and their families, for example focusing on the impact of visitor bans including specifically on residents with dementia [6–10]. Another important focus was placed on the experience of staff working in nursing homes, especially front-line nurses but also NH administrators and personnel in leadership positions. Studies reported a significant negative impact of the pandemic on staff in terms of mental wellbeing, stress, burnout or moral injury, increased workload and staff shortages, as well as being caught between protecting their patients’ physical and mental health as well as their own [11–15].

In Germany, several surveys reported pandemic-related staff shortages and increased workload, financial challenges in light of protective equipment and regular testing of residents and staff, the (non-) uniformity of guidelines throughout Germany, and occupational heat stress experienced by staff wearing protective equipment in the summer of 2020 [16–18]. A qualitative study reported similar findings, and also reported the reorganisation of physical spaces (e.g. quarantine stations), the implementation of hygiene measures, increased communicational demands and conflicts between staff and residents, and more emotionally demanding care work during visitor bans. This included caring for residents with dementia, psychological stress and the burden of responsibility [19]. Another study from the German context reported that residents, relatives and staff sometimes reacted with verbal violence to the infection control measures, but that this was not perceived as a major concern by the questionnaire respondents (nurses) [20].

Most studies into NH experience were conducted at the height of the COVID-19 pandemic, especially from 2020 to 2021, when insights into this setting were acutely needed. We aim to add to this body of research by providing insights from a timepoint when the acute phase of the pandemic was over and measures had been rolled back, enabling staff to look back and reflect. This study aims to answer the following research question: Looking back after the pandemic, how did NH staff subjectively experience the pandemic at their workplace and which lessons learned do they draw for the future?

## Methods

### Research design

We conducted a qualitative study with semi-structured interviews. The institutional review board of the Medical Faculty at Goethe University Frankfurt approved the study (2022 − 968). All participants provided written and verbal informed consent before participating in the study. In reporting our research, we follow the Standards for reporting qualitative research [21].

### Setting and team

The study was conducted at three nursing homes in the federal state of Hesse, Germany (NH-A, NH-B, and NH-C). The core team responsible for data collection and analysis consisted of two students with health professional backgrounds: DW, a dental technician and Master student in Public Health, and AKM, a nurse and medical student, both supervised by LB, a social scientist with > 10 years of experience in qualitative and health services research. None had any prior contact with the case sites.

### Recruitment and participants

The three NHs were sampled from an existing sample of NHs that had previously cooperated with the Frankfurt Institute of Medical Virology, and which were asked to participate in our current study [22]. Within each nursing home, we aimed at a heterogeneous sample of staff who had been working at the NH during the COVID-19 pandemic. This could include current employees, former employees, employees who had worked at the NH during the entire pandemic, employees who started working at the NH during the pandemic or employees who worked at the NH during the pandemic as part of their training. Preparatory steps were conducted with NH leadership to explain the purpose of the study as well as the sampling strategy. It was emphasised that the researchers wished to speak with a diverse group of staff, in terms of age, gender, experience (in general and during the pandemic), job type and level, job satisfaction (i.e. that the aim was not to only hear about positive experiences). NH leadership then provided an initial list of suggested interviewees that was discussed with the research team. At each site, NH leadership was instrumental in scheduling interviews in such a way that was least impactful for staff’s work schedules and allowed staff to conduct interviews within their paid working time.

### Data collection

Data collection took place between May 2023 and May 2024, first at NH-A (May 2023), followed by NH-B (November to December 2023) and NH-C (April to May 2024). The interview guides were developed based on a preliminary literature search conducted within the scope of DW’s Master thesis. In the preparatory phase, we consulted with a German politician with a background in nursing and piloted the interview guides with a nursing director who was not part of the current study sample. Both stakeholder informants provided relevant context information from the nursing perspective and provided the students with an opportunity to practice their interview skills. Throughout the data collection phase, the interview guides were regularly evaluated by the research team and adapted where necessary. For example, topics such as “digitalisation” were given less importance over time, as interviewee responses tended to be similar and no new or more relevant data could be gathered. The topic of vaccines / vaccine mandates was given more importance over the course of the study as researchers gained more confidence in approaching this complex topic (as indicated in Table 1, this will be reported in more detail separately).

The main dimensions with sub-topics are shown in Table 1. Most interviews were conducted on-site and in person, except when the interviewee asked for an interview by video-call. Demographic data was collected on gender, age, professional qualification and/or current profession, and years of work experience (see Table 2). All interviews were conducted in German.

**Table 1:** Interview guide (overview; translation from original German)

Topics	Sub-topics
Introduction	• Description of typical working day before pandemic • Beginning of pandemic • Remembering beginning of pandemic • Impact of pandemic on work, NH, daily routines
Organisational matters	• Availability of resources (e.g. masks, tests, protective equipment) • Contact with regulatory agencies / government • Implementation of laws and regulations • Regulations regarding testing • Digitalisation
Communication	• Internal information flow regarding changes, e.g. new laws • Subjective impression of (not) feeling well-informed • Other (external) information sources used by interviewees
Working conditions	• Hygiene measures; implementation, subjective evaluation • Physical and psychological burden • Contact with relatives • Resident deaths during pandemic • Potential impact of vaccines, views about vaccines in NH* • Potential staff lay-offs during pandemic
Residents	• Interviewees’ impression of residents’ experience of the pandemic • Atmosphere during pandemic
Personal life	• Impact of pandemic on personal life (outside work) • Own COVID infection / illness, recovery • Resources and challenges to deal with problems and worries
Conclusion	• Pandemic changes (in NH) that still exist • Lessons learned • What could or should have been done differently • Wishes from politicians and/or other people • Feeling of (un-) preparedness for next pandemic • Looking into the future
Open question (asked twice)	• Is there anything you would like to mention that we have not mentioned so far?

Notes: *Given the complexity of this particular sub-topic, findings related to vaccine attitudes and behaviour are analysed and reported separately (not part of the analysis of this publication)

### Data analysis

Interviews were audio-recorded and transcribed verbatim, using the transcription software Vink [23] for initial transcription, followed by revision and quality checks. Data analysis was conducted using reflexive thematic analysis by Braun and Clarke, following the process described in Braun et al. 2023 [24]. Specifically, this included the six phases of (1) familiarisation with the dataset, (2) coding, (3) generation of initial themes, (4) development and review of themes, (5) refining, defining and naming themes, and (6) writing up the report. Whenever possible, we chose to use the interviewees’ own words, especially when they transported a meaning that transcended their own individual experience. Interview quotes were translated from German to English and shortened where necessary for brevity and clarity.

## Results

### General results

Interviews were conducted with 41 staff members in three nursing homes. Interviewee characteristics are summarised in Table 2. The following main themes were developed by the research team: (1) “It’s us against the world”; (2) “Was it the physical or psychological burden? – Both”; (3) “Death and dying – but not in these quantities, not in these circumstances”; (4) “From absolutely unprecedented to practiced routines”. A fifth topic, COVID-19 vaccination attitudes and behaviours of staff, is to be reported separately.

**Table 2:** Summary of interviewee characteristics

Interviewee characteristics	Number	Percentage (%)
**Gender**		
Male	7	17,1
Female	34	82,9
Diverse	0	0
**Age** (in years); average 42,9 years		
19–24	1	2,4
25–29	6	14,6
30–39	11	26,8
40–49	11	26,8
50–59	7	17,1
≥ 60	5	12,2
**Qualification / Profession** (more than one category per interviewee possible)		
Nurses*	29	70,7
Leadership and managerial positions**	20	48,8
Administrative staff	4	9,8
Therapy, social work & daily activities	6	14,6
Other (e.g. cleaning personnel)	2	4,9
**Work experience** (in years); average 13,2 years		
1–2	2	4,9
3–5	7	17,1
6–10	11	26,8
11–20	14	34,1
≥ 20	7	17,1

Notes: *including qualified nurses (Pflegefachkraft), nursing assistants (Pflegehelfer*in), student nurses (Pflegeschüler*in); **including care home managers (Einrichtungsleitung/Residenzleitung), care managers (Pflegedienstleitung), area managers (Wohnbereichsleitung), quality managers (Qualitätsbeauftragte*r)

### Theme 1: It’s us against the world

Most interviewees emphasised the great team spirit, solidarity, cohesion and resilience among staff members never experienced before. Interviewees reported how they had leaned on each for support through the worst, mainly by talking and lending each other an ear. Another specific way of supporting their co-workers was through working – working more, taking on additional shifts or returning early from holidays and sick leave, often at the expense of one’s own wellbeing.

*I drove back [from holiday] and I worked through New Year’s. Because this team spirit… You couldn’t stay home. I couldn’t stay on the sofa. NH-B, Interview 5*

*Some of my colleagues*,* I have such great respect for them. Even though they were [COVID-] positive*,* mild symptoms*,* they came to work (…). I find that great*,* great (…). NH-B*,* Interview 3*

*Theoretically*,* I was supposed to stay home during the school block. But when you hear and see that there are just two [nurses]*,* instead of six (…)*,* I went to work. PNH-A*,* Interview 1*

In contrast, co-workers perceived not to be pulling their weight, e.g. by calling in sick, were described negatively. While many interviewees did report understanding for their co-workers given their or their families’ health risk factors, they did still also voice feelings of resentment, as they felt it to be at their own expense that others were spared.

The strong social cohesion at work was named by many interviewees as a remedy to the loneliness resulting from the restrictions of the pandemic, stating that they were happy to be allowed to see their colleagues at work, instead of being isolated at home. However, this also meant that the strict hygiene and protective measures implemented at the NHs negatively impacted their ability to draw strength from each other.

*I was never as alone as I was during the pandemic. I was just happy to have a job like this*,* which allowed me to go outside every day. That was the only thing that made me happy: to go to work (…). NH-A*,* Interview 6*

*Many things suffered under these strict measures. (…) Only one person was allowed in the nurses’ office. One in the front*,* one in the back*,* but not together*,* not even with a mask*,* you [had to maintain] distance. You couldn’t take breaks together as a team*,* no matter how big the room was. You couldn’t go outside together to smoke*,* always alone. NH-B*,* Interview 10*

Many interviewees described the experience of working during the heights of the pandemic as being “on an island, in a cocoon”, (NH-B, Interview 4), “like a parallel world“ (NH-B, Interview 4), “a microcosm” (PNH-C; Interview 3) which they contrasted with the outside world, including partners, friends, family and society in general. Some interviewees thought that “outsiders” had trivialised the pandemic, behaved more carelessly or would forget more easily now that the worst parts are over.

*Recently*,* a friend’s father told me that COVID never existed anyway. I told him: [Horst]*,* I’ll slap you in the face. NH-B*,* Interview 2*

*[People said]: It’s all nonsense. […] No one is dying. I said: You can only say that because you did not experience it. I experienced it all. Whether or not you believe it*,* it did happen. NH-B*,* Interview 3*

*I mean*,* how could they know*,* if they haven’t lived through it? NH-B*,* Interview 4*

Some interviewees would have wished for more compassion from others, sometimes issuing invitations for “outsiders” to walk a day in their shoes and experience their realities. The latter was sometimes addressed specifically to politicians responsible for the decisions affecting the conditions at NHs during the pandemic.

*Sometimes*,* when I would hear things*,* like on the bus*,* […] I would tell them: you can come visit us and see how it is to be isolated*,* or to care for someone who is infected*,* who is really weak. [Let’s see]*,* if you still think it doesn’t exist. NH-B*,* Interview 16*

*[The politicians] always do things [but] no one asks [us]! Come here*,* why don’t you*,* to a place which experiences it every day. NH-A*,* Interview 5*

When asked about the future, many interviewees indicated their positive experiences regarding the strong team cohesion as a significant source of strength and optimism for future challenges:

*[My colleague] and I were new here. We told each other: if we make it through this*,* we’ll make it through anything. NH-B*,* Interview 2*

*With the team that we have here […]*,* we’d make it through another extreme situation like this. NH-C*,* Interview 13*

### Theme 2: Was it the physical or psychological burden? – Both

#### Physical burden and workload

Interviewees from all three NHs described the exceptionally high workload during the pandemic, principally due to the high disease burden and resulting higher care burden for infected patients, and the extensive hygiene, isolation and protective measures to prevent outbreaks.

*We worked like crazy. We sweated*,* we cried*,* we worked like idiots. We were exhausted to the bone. NH-C*,* Interview 6*

*I just functioned*,* because I didn’t even have time to think. […] I worked*,* worked*,* worked. Sometimes 10 hours a day*,* or from the early shift to the late shift*,* because there was no staff. NH-B*,* Interview 13*

The physical work was experienced as more physically exhausting due to the protective equipment (e.g. gowns, masks), especially during hot summer periods. The more frequent patient deaths also incurred additional workload, for example because new patients had to be admitted more frequently, causing additional stress for administrative workers and nurses.

All this was exacerbated by severe staff shortages due to illness as well as COVID-positive staff being prevented from returning to work during earlier periods of the pandemic.

*[They fell] like dominoes*,* one after the other positive*,* positive*,* positive. Within a day*,* maybe three or four people were positive and they had to be sent home. PNH-C*, *Interview 6*

*You would use all the positions*,* all the staff you have (…). At a certain point*,* when there was no more staff*,* you would gradually minimise [the level of] care*,* (…)*,* I’ll call it (…) “fed and clean”. NH-C*,* Interview 13*

*When there just was no more staff*,* […] then you would be responsible alone for 18 [COVID-] positive patients on a ward. And if you were unlucky and there were more absences*,* you’d be responsible for even more. NH-B*,* Interview 9*

In addition, NH staff had to carry out additional tasks that did not exist before. Examples include mandatory and large-scale testing of residents, relatives and staff members, establishing and maintaining isolation wards. It also included administrative measures such as communicating with and keeping extensive lists for different authorities (e.g. of infections, test results, vaccination status, etc.) and informing staff of new regulations. Other new tasks included planning and supporting appointments or video calls with relatives.

*The health authorities wanted lists of the lists of the lists of the lists. But not only the health authorities*,* also [our headquarters] wanted lists (…): where did all the tests go? So you would write the lists of the lists of the lists*,* basically from morning until evening. NH-C*,* Interview 13*

*Twice a week we had to test all residents. (…) Testing everyone*,* so changing your gloves every time*,* changing your mask. For the positive [residents]*,* you had to change your clothes completely. (…). I would spend almost 5 or 6 hours testing entire wards. NH-C*,* Interview 12*

#### Psychological burden

In addition to the high physical burden, staff also reported a high psychological burden experienced during the pandemic. This was due to factors such as the unusually high frequency of patient deaths during outbreak waves, their own worries and fears about infection (their own, their loved ones’ and the residents’), and the uncertainty of the pandemic situation.

*I would have preferred to (…) stay home. But I can’t*,* […] there is no staff. These fears (…). Why should I go to work? To risk my life […]? But what am I? I am worth [something] too. (…) The fear and the responsibility are present at the same time. But the responsibility is bigger. (…) I was afraid to go work. I was afraid to infect my husband. NH-B*,* Interview 13*

*I told [NH leadership]: I’m not in a senior role to die miserably in this NH. […] It’s still just my job*,* not my life’s mission. But my sense of responsibility drove me there every morning. NH-B*,* Interview 9*

Other work-related stressors included feelings of guilt for outbreaks, especially at PH-B, the feeling that the pandemic just kept dragging on over months and years with no end in sight, not being able to carry out their normal rituals or just take time to mourn for the residents they lost, and the feeling of not being supported or valued by the authorities.

*What is the priority of this stupid list in an emergency situation of a global pandemic? Because the insurance does not want to cover three tests? That’s nice. Who reimburses us for our emotional damage that we incurred? No one cares about that. NH-C*,* Interview 1*

Some interviewees also reported complaints, including insults and aggressions, from residents and relatives, which also weighed negatively on them.

*There were these quarantine times when residents had to stay in their rooms. Then there was a lot of trouble with residents*,* who yelled at us*,* threw things at us*,* told us they were going to call the police. […] There was so*,* so much mentally*,* that was so exhausting. NH-B*,* Interview 9*

*When the NHs closed their doors*,* [relatives] threw flowerpots into the windows*,* yelled at us*,* secretly climbed over the fence*,* banged at the windows like crazy people. NH-C*,* Interview 4*

It was not only the work-related experiences, but the personal situation as well. Not only because there was much less personal time left, given the long hours and the severe exhaustion experienced after work, but it seemed to many interviewees that there was no escape from the pandemic anywhere, in addition to the isolation and restriction of normal activities due to the social regulations.

Most interviewees reported that there had not been any offers of psychological support by their employers, for example in the form of external psychologists or counsellors, anything beyond the support they received by colleagues or leadership. NH-B was an exception, as it had experienced a devastating outbreak, and staff were able to talk about their experiences in a supervised setting. While some interviewees appreciated psychological support, or would have wished for such an offer, many interviewees indicated that they did not or would not make use of it because they preferred to deal with their experiences either on their own, within their team or their personal support system.

### Combination

In general, it was very common for interviewees to describe aspects of the physical and psychological burdens they experienced in combination, highlighting their interrelatedness and how one amplified the other, or decreased their ability to cope with challenges.

*It was so much work and it was exhausting. Back then*,* we had staff shortages and (…) we had to take care of both wards. (…) We had an isolation ward*,* where only one person per shift was allowed to work. (…) And in the summer*,* too*,* with the protective equipment (…). It was a disaster. And the [staff] were so nervous as well. (…) It breaks you physically*,* and […) it breaks you mentally. NH-C*,* Interview 7*

*Interviewer: “Was it exhausting just for your body*,* or also for your head?”*

*Interviewee: “Both. It was both. (…) It all comes together (…) We have to finish everything. (…) We worry like “I still have to do this*,* I still have to do that.” To think like that and the pressure; it destroys your head.” NH-B*,* Interview 11*

Another example of this interrelatedness was the complicated and time-consuming implementation of the protective measures regarding patients with dementia. Staff reported that no matter how much they tried, these patients could not understand and follow the rules, and that they felt many measures impossible to implement in good conscience.

*I would like to tell you about the (…) worst experience of the pandemic. It’s about the [COVID] tests that were decreed*,* that [we] had to do [against residents’ will]. Those are things I still dream about until this day. […] I don’t know if it’s so great to kneel on someone with dementia*,* kneel on their shoulders*,* so that they cannot defend themselves. One [nurse] fixes their head*,* so I can take the swab. That was one of the moments when I wanted to call the authorities: “come here and do [it] yourself.” I don’t want to look into those teary eyes that look at me full of panic and ask: what do you want from me? NH-C*,* Interview 1*

### Theme 3: Death and dying – but not in these quantities, not in these circumstances

Many interviewees from all three NH emphasised the significant quantitative difference in resident deaths during the pandemic and during specific outbreak waves. While they explained that death and dying is a normal part of living and working in a NH, they pointed out that the pandemic experience differed starkly from what they would usually handle within their professional experience.

*The first wave*,* when we had so many deaths at the same time (…). It was eerie (…) because you got an inkling of… here’s something that didn’t exist before. (…) Suddenly*,* so many people were severely ill and dying*,* you would get the feeling: something unknown is coming. NH-A*,* Interview 11*

*Of course*,* residents die in nursing homes. (…) But not in this quantity*,* not in these circumstances. NH-A*,* Interview 7*

*(…) Within the next days*,* another resident would die*,* two*,* three. And the next day again. (…) Almost half of our residents died. (…) Sometimes I didn’t remember who had died. NH-B*,* Interview 13*

*That was the first time I saw that there are undertakers who can fit six coffins into a hearse. NH-B*,* Interview 9*

Some interviewees pointed out that residents died more unexpectedly than in non-pandemic times, meaning death occurred more suddenly, more rapidly, and in residents who were not expected to pass away so soon. In addition to the significant psychological impact, interviewees also described that these deaths (and therefore, new admissions) caused significant additional workload. For NH leadership, there were also financial questions as rooms were blocked for a period of time after resident deaths, and new residents could not move into NHs during outbreaks. Due to pandemic regulations, it was also often not possible for staff to follow their usual rituals to help them to cope, such as tidying up the room, dressing the resident nicely, or going to funerals to say goodbye.

*It’s important to me that we are allowed to go to funerals again. (…) I need a good-bye. (…) There are residents who’ve become dear to you over the years (…)*,* and I would like to go the last steps with them. NH-A*,* Interview 6*

Many interviewees reported that given the high workload and severe time constraints, they did not have enough time to support residents’ dying process. They perceived this as emotionally burdensome, especially when also thinking about the fact that in the very early phase of the pandemic, relatives were not allowed inside the NHs, and some residents died alone.

*We are used to coping with death and dying*,* but somehow*,* it’s a different way of dying. If you care for someone palliatively over many weeks and months*,* that’s a nice way of dying*,* a nice way of accompanying [someone]. If I find someone and they are just lying dead in their bed*,* that’s (…) worse for us. It was bad. […] There was a lot of crying. NH-A*,* Interview 5*

*It was not easy. (…) I lost one resident. He died very*,* very suddenly. And I [still think]: If I hadn’t been alone with 12 [residents]*,* maybe I would have had more time*,* or maybe I could have helped him. NH-B*,* Interview 7*

*It took a long time*,* looking back [now] (…)*,* until it was possible for relatives to visit their dying [residents]. (…) We had one or two cases*,* which still weigh on me personally*,* where I know that someone was alone [during] their last days. […] I could have gone in [to the room]. But we tried to have as little contact as possible*,* which is completely crazy*,* because if someone is dying*,* what is supposed to kill them? NH-B*,* Interview 1*

### Theme 4: From absolutely unprecedented to practiced routines

From all three NHs, many interviewees reflected on the unprecedented nature of the situation in which they found themselves. Many were able to recall in detail the specific date or event that marked the start of the pandemic at their NH. They emphasised that not only did they not know what to do – no one else did either. There was no plan, there were no guidelines, and no one to ask for advice.

*(…) No one knew how to deal with it. No one. (…) Are the relatives allowed inside*,* are they not? Are they allowed into double rooms*,* with more than one relative*,* or not? It was absolute chaos*,* because no one could tell us anything. And these state orders (…)*,* it felt like there were five new ones per week. All to be implemented immediately. Friday 4pm*,* here you go*,* implement it. And you wonder: Who? With whom? With which material? Why? And what do I tell the relatives? NH-C*,* Interview 13*

*Do I have to disinfect the [food] cart before it goes back into the kitchen? Because no one knew. If this food cart is in the ward kitchen for an hour … is it contaminated? (…) And then you take the food cart into the [main] kitchen (…) and if the main kitchen is out*,* that would be the absolute worst-case scenario. NH-A*,* Interview 7*

In this atmosphere of uncertainty, interviewees described that new regulations from the government or other relevant authorities were often perceived to be ever-changing and unclear. Consequently, interviewees described how they, within their NH, within their teams and leadership, tried to make sense of official regulations. Some interviewees described instances when they themselves or their NH decided to deviate or act independently. This happened especially when regulations were perceived to be nonsensical, too burdensome given workload and staffing, not implementable (e.g. with patients with dementia) or ethically indefensible.

*[In these cases,] we did not follow the current law and regulations. Because it’s all nice and well but at the end of the day*,* I’m still a nurse. And I still have someone in front of me who was entrusted to my care. They’re not an animal*,* they’re not a machine*,* they’re a human being with human dignity. And part of human dignity is to not die alone. [Nurse]*

*[…] Every authority wanted something different. I told them: […] [No.] One form*,* I send it to you in the evenings*,* you don’t get anything else. I don’t document*,* I don’t write […] reports*,* I won’t do any of that. If you want to know how we work*,* come here and have a look. […] [Our] computers were turned off. [NH leadership]*

The unprecedented nature of the pandemic was sometimes cited as a reason why interviewees did not blame themselves or others for mistakes or decisions that turned out to be wrong in hindsight. When asked about future challenges or changes over the course of the pandemic, many interviewees from all NHs pointed out how much they had learned and how much expertise they had acquired. This gave them confidence with regard to new outbreaks or potential future pandemics.

*It was noticeable during the second outbreak (…). With the first positive [resident]*,* [my colleagues] knew exactly what to do. […] They notified*,* made lists*,* tested*,* got protective equipment. They knew how to work with it. They had learned from the first wave. NH-B*,* Interview 6*

This confidence in their acquired expertise led several interviewees to ask why no “outsiders” asked them for advice (e.g. politicians) or to question the authority of those agencies who had stayed away from the NHs during the pandemic, and now started to check on them again. Instead, they felt that they had proven their worth and ability during the pandemic and should be awarded more trust and autonomy in return.

*The supervisory authorities try to [go] back to normal*,* but for three years [they] didn’t care at all how we managed. […] We saw that when push comes to shove*,* you don’t need to tell a nurse anything about hygiene. We know what we have to do. We know. We have the images. That’s what I always told the supervisory authorities: You don’t have to tell me anything. You have the names and the dates of birth. I have the image behind that*,* and the relative. This chasm is more extreme than it was before. Between those people who have lived through it*,* and those who now want to tell us how to do our jobs. [NH leadership]*

## Discussion

This paper investigated the experiences of the pandemic from the perspective of staff working in nursing homes. Four themes were developed which shed light on aspects of teamwork, workload and wellbeing, resident deaths and preparedness for future challenges.

### Summary and discussion of results

Theme 1 “It’s us against the world” refers to the strong sense of team spirit, mutual understanding and working towards a common purpose reported by staff in all three NHs, from which they drew strength in hard times and optimism for future challenges. Often, this was contrasted against the outside world of relative normality and of those who had not lived through the same experience. Closer team cohesion was also reported in other studies [25], often specified as a main facilitator enabling NHs to persevere through unprecedented challenges and as a source of confidence and resilience [26, 27]. Like ours, other studies also reported staff relying on each other for psychological support, sometimes in combination with (demand for) outside resources [26, 27]. Burton et al. used the term “compassionate teamwork” to describe several of these qualitative changes in how staff related to each other [28].

Theme 2 “Was it the physical or psychological burden? – Both” describes the co-incidence of an exceptionally high pandemic-related workload in combination with critical staff shortages, with a high psychological burden, related to both the situation at work as well as at home. Crucially, the two burdens were experienced as related and intertwined, one often amplifying the other. The high and increased workload (sometimes called overload) in combination with severe staff shortages were reported in many other studies [26, 28–34] including studies conducted in Germany [16, 17, 19]. One German study specifically reported on occupational heat stress experienced by nurses working in protective equipment on hot days [18], as was also described by our respondents. Many other studies additionally found an important negative impact on staff psychological wellbeing during the pandemic, due to a range of factors including stress, fear of infection (including severe disease and death), or the responsibility to not make a mistake potentially causing an outbreak [14, 27, 28, 30, 32–34].

Altintas et al. summarised many of these aspects with the term “emotional exhaustion”, which also seems a fitting descriptor of our findings [35]. One German study reported that residents, relatives and staff sometimes reacted with verbal violence to the infection control measures, but that – in contrast to our findings – this was not perceived as a major concern [20]. As can be observed from overlap in references cited for physical and psychological burden, their co-incidence was reported by many others as well.

Theme 3 “Death and dying – not in these quantities, not in these circumstances” summarises the staff experiences of residents’ deaths and dying during the pandemic, both in quantitative terms, i.e. unusually many residents dying in a short period of time, as well as qualitative terms, e.g. residents dying unexpectedly, without their relatives, and staff not being able to support the dying process or to mourn the dead as they would usually do. These aspects were also reported in other studies, which also emphasised the stark differences to normal times, which include dying as a normal part of living and working in NHs, as was also described by our respondents [25, 28, 36–38]. Rutten et al. mentioned that the restrictive measures prevented other residents from saying their last goodbyes to dying residents, which seems likely to have also happened in our NHs, but which was not mentioned by our staff interviewees [25].

Included in Theme 3 as well as Theme 4 are aspects of complex ethical and moral decision-making addressed by the interviewees, for example when they felt pressured by laws or regulatory authorities to implement rules or perform tasks they experienced as unethical or immoral, or when they perceived that no “good” option was available, for example when protective measures had an isolating effect on residents or when insufficient staff was available to provide adequate care. These experiences have also been described in other studies, often as moral injury, moral wounds or moral suffering [39–42]. In our study, these were described by interviewees as their individual experiences, but generally not framed within a larger concept or given a specific name, such as the ones used in the published literature.

Theme 4 “From absolutely unprecedented to practiced routines” entails the development from a situation in which no one knew what to do and expertise had to be gained through lived experience, to a situation of relative calm, experience and expertise from the perspective of NH staff. This was often linked to calls for more respect and autonomy as well as for “outsiders” to listen and learn from NHs. Many studies also highlighted the unprecedented nature of the situation in which staff found themselves [43], leading to learning experiences and coping and adaptation strategies developed by staff [2, 28, 38], but also the lack of or tension with external guidelines and the wish for more support, including from management and regulators [14, 28, 44, 45]. Referring to the disaster response in Italian NHs, Plagg et al. (2022) concluded that the unprecedented “shift from resident-centered care towards collective-protective approaches led (…) to an emergency vacuum” [46]. Aspects of such an “emergency vacuum” were also reported by our sample, but generally, this seems to have been mitigated to a certain extent by team cohesion and NH leadership support and decision-making, protecting staff from the worst.

### Lessons learned and recommendations for future scenarios

Given the findings reported in this study, there are several recommendations for improvement that can be considered for future pandemic or larger outbreak scenarios. First, it seems a promising approach to prioritise strengthening team cohesion as a resource to be able to rely upon in future emergency situations. In many reported instances, a strong team spirit and reliance on co-workers helped turn a challenging experience into a manageable one, allowing for personal and professional growth and confidence in the ability to face future challenges. Team cohesion and peer support also hold important potential for resilience against the considerable psychological burden reported by many interviewees. However, NH should make sure to protect their staff when team members expose themselves to overwork and increased physical burden to support their co-workers especially in times of staffing shortages, as was also reported in our study.

Given the perceived insider-outsider divide related to the pandemic experience, it may be promising to consider training psycho-social support workers from within the field and within a given NH, rather than bringing in outsiders. Such peer support workers who also care hands-on for residents seem likely to receive more trust and respect and be able to offer more understanding to fellow frontline workers than a (perceived) outsider. This would also offer a practical solution to the problem of outsiders not being allowed to enter NHs during active outbreaks or while certain pandemic regulations are in place. Such an approach would require more flexible staff-, skill- or task- mix solutions within NHs, to ensure that staff can be trained and work in additional functions while still remaining at the frontline. Such a more flexible staff-, skill- or task- mix approach could also be considered for knowledge pipelines from within to outside the NH. A frontline worker with additional training and responsibility for liaising with the outside world could thereby facilitate the flow of insider knowledge and experience from the frontlines to “outside” agencies responsible for governing “inside” realities.

Finally, these aspects could also be considered in a temporal dimension. Many societies and health systems are now in a more advantageous situation than before COVID-19-pandemic: where there was expertise and experience regarding outbreak prevention (e.g. of influenza or norovirus), there was not of a global pandemic. There is now a significant number of experts who have lived through an active pandemic and who have relevant knowledge and training not only related to pathogen and hygiene measures, but also to how an NH switches to pandemic mode, e.g. how they upheld communication or how they tried to motivate their staff. The first-hand knowledge of these “experts by experience” from the Covid-19 pandemic could be intentionally harnessed and maintained, not only in the form of outbreak manuals, but also in the form of experienced guides and peer support workers for future generations of health professionals.

However, there are important potential complicating factors that should not be overlooked when conceptualising future efforts, as they play a crucial role in whether these efforts can be implemented successfully, or at all. First and foremost, all efforts envisaged here would cost money and require personnel – but how to achieve this when the money and staff were not even sufficient for minimally required patient care during the pandemic; and staffing shortages were already a salient issue before the pandemic. When would the staff responsible for patient care make use of the peer support systems when they were overwhelmed with an unprecedented workload? Moreover, making first-hand experiences of the past pandemic available to future generations would require that those holding the experience remain in the nursing profession for the foreseeable future. Similarly, if a society wishes that pipelines are established from insider knowledge to outside actors, e.g. supervisory authorities and policy-makers, this should involve realistic facilitating measures such as reimbursements and infrastructural measures so that NHs and nurses wishing to share their expertise do not need to worry about leaving residents without care or abandoning their colleagues to more work. Moreover, as is described in the limitations sections, we assume that we likely had NHs in our sample with more advantageous staffing situations before and during the pandemic, than those who did not participate in our study. We do not know whether strengthening team cohesion or training peer support workers in those cases would achieve the same benefits as reported in our study or whether this is only a promising parameter in NHs with a good baseline staffing level. For example, if staffing levels are so low that no team building efforts can be conducted without endangering residents or overworking personnel, or if trust between staff is eroded to an unmanageable extent, other efforts are likely needed more urgently.

All of this underscores this importance of implementing most improvement efforts in pre-, rather than pandemic times, as an emergency situation is unlikely to allow for extensive relationship building or training investments other than those strictly related to the pandemic response. This is to say that pre-pandemic conditions matter for a good pandemic response, as they are the foundation on which the emergency response is built. This includes both negative conditions, such as staffing shortages, high workload and frustration or mistrust towards regulatory agencies or politicians, as well as positive ones, e.g. resilience factors such as team cohesion and robust leadership. Finally, this means that at a certain point, the question of future (pandemic) preparedness moves from a scientific question to a policy matter. When sufficient studies have underscored the importance of staffing levels and staff satisfaction in various health care settings, including NHs, and in various contexts, including pandemics, the decision to prioritise, fund and implement improvements measures then lies with the responsible policymakers.

### Strengths and limitations

Our study has several limitations to be taken into consideration. It is likely that selection bias played a role both in the selection of NHs and interviewees. While NHs were contacted from an existing larger sample, the three participating NHs self-selected from within this group. Two additional NHs declined to participate citing time constraints and high workload of the nursing staff as the main reasons. We therefore assume that our three NHs may be in a somewhat more advantageous position (e.g. in terms of staffing) than average and therefore may have had on average more positive experiences during the pandemic than those that did not participate. Within the NHs, sampling of interviewees was prepared and conducted in a stepwise approach, informing NH leadership of the sampling strategy and aim. Still, the final composition of the participant group was significantly influenced by NH leadership, and we cannot know whether relevant perspectives may have been missed. While we were able to include one interviewee in our sample who quit their job during the pandemic, we were not able to include more of these experiences in our study, which could have provided additional and different data. We do know that our results include negative staff experiences and criticism of NH leadership as well as positive ones. In combination with the impact of NHs selection, it seems likely that our results do not reflect the worst experiences of the pandemic. Moreover, as we conducted our study at the end of the pandemic and over a period of approximately a year, we were concerned about re-call bias. However, this did not seem to be the case, especially for the first phase of the pandemic and those issues that were important to the respondents. Interviewees were able to re-call in great detail, for example the first days of the first outbreak, the first positive patients or how specific dying patients looked. Memories did seem to fade regarding when which regulation changed, or with regard to regulations that were in effect only temporarily. Even though our study aimed to add to the body of literature by providing an additional perspective from after the active phase of the pandemic, the comparison of our results with the literature from active phases shows more overlap than we would have expected. Our study does add new insights especially as reported in theme 4, with regard to staff’s confidence in their preparedness for future pandemics and possibly also with regard to looking back with less blame for mistakes, which we saw reported less in studies conducted during active phases. Finally, it should be noted that our sample only includes NHs from the German federal state of Hesse, meaning that there may be limits to the transferability to other states.

The main strengths of this paper include the heterogeneous participant group, e.g. in terms of professional background, hierarchical level, age, and work experience. Our sample also includes nurses with immigrant experiences, providing additional insights given the international scope of the pandemic. Finally, NH leadership allowed their staff to be interviewed during paid overtime, making it possible for busy staff to take time for the interviews and speak in detail about their memories.

In terms of future research, it would be helpful to conduct more mixed methods research in NHs to be able to combine the advantages of large-scale surveys with the detailed, in-depth insights of qualitative research. In the order of an exploratory sequential design (i.e. QUANT following QUAL), this would allow for an in-depth exploration of salient issues from the interviewees’ perspective, and then to investigate to what extent these are reflected within larger, or even representative, samples. As many similar NH studies were conducted during the height of the pandemic, it would be helpful for researchers to document in detail about how they were able to conduct research in these emergency situations, especially in vulnerable settings, so that research as well can switch more easily to a pandemic mode in the future.

## Data Availability

The interview transcripts are not publicly available due to ethics requirement and to protect interviewees’ identities. Data excerpts can be requested from the corresponding author on reasonable request.

## Abbreviations

NH: Nursing home
PI: Principal investigator
